# One Gene, Two States: Truncating Loss Versus Retained Full-Length p53 in Solid and Hematologic Cancers

**DOI:** 10.3390/ijms272110024

**Published:** 2026-11-09

**Authors:** Haichun Huang, Yikui Xie, Baoping Guo, Ying Duan, Hong Cen

**Affiliations:** Department of Hematologic Oncology, Guangxi Medical University Cancer Hospital, Nanning 530200, China

**Keywords:** TP53, truncating mutation, loss of function, mutant p53, retained full-length protein, mutation spectrum, allele-specific therapy, clonal hematopoiesis, hematologic malignancy

## Abstract

TP53 mutations create two functionally opposite states—loss of the protein or retention of a full-length mutant protein—yet are usually reported as one entity. How the two states are distributed between solid tumors and hematologic malignancies has not been quantified. In one pipeline integrating ≈64,000 samples (29 solid-tumor types, 19 hematologic cohorts and a 42,714-sample post-therapy clonal-hematopoiesis cohort), we asked which state is selected where, what it does, and what it offers. Truncating mutations accounted for 34.2% of solid but 24.4% of hematologic alterations (χ^2^ = 33.7, *p* = 2.3 × 10^−7^; reproduced in the NCI TP53 and COSMIC databases). Where copy number resolves allelic state, 60.6% of missense-mutant solid tumors had lost the wild-type allele (19.5% biallelic inactivation). Blood retained full-length missense protein instead, and in MDS, outcome tracked the retained-allele dose (hazard ratio 1.78, *p* = 1.0 × 10^−4^); in solid tumors, no survival difference was detected by allelic state. The endogenous CpG baseline is equivalent (20.7% versus 21.2%), while solid spectra carry exogenous exposure fingerprints. Only the retained state presents a target, so allele-specific reactivation is the rational corollary where retention prevails—a hypothesis, as retention in blood is inferred from mutation class.

## 1. Introduction

TP53 is the most frequently mutated gene in human cancer, and its mutations fall into two functionally opposite states. A truncating mutation (nonsense, frameshift or splice-site) destroys the protein and is the most direct route to loss of wild-type p53 function. A missense mutation instead yields a full-length product that, in many but not all cases, retains the DNA-binding architecture while acquiring dominant-negative and gain-of-function (GOF) activities [1]. A substantial fraction of missense mutants are destabilizing or functionally null, so “full-length” here describes preservation of the coding sequence rather than proven stability or activity. The two states, therefore, differ not only in degree but in kind: one removes a tumor suppressor, and the other installs an oncogenic protein. That asymmetry has a therapeutic corollary, because only a retained full-length protein can be targeted by allele-specific reactivation or by reagents that recognize mutant peptide–HLA complexes, whereas a protein that is absent cannot (Figure 1). Loss of the wild-type allele does not remove this substrate: the mutant protein is still made, so it remains targetable even when the wild-type copy is gone.

Which of the two states a tumor carries is not a matter of chance. It depends on what writes the spectrum and on what the tissue tolerates. Solid-tumor spectra are read as records of carcinogen exposure—ultraviolet-light C→T transitions at dipyrimidine sites in skin cancer [2], benzo[a]pyrene adducts in lung cancer [3], aflatoxin-driven R249S in hepatocellular carcinoma [4]—and, in that compartment, loss of the wild-type allele is a routine event, so complete inactivation is cheap. Hematologic spectra are written instead by endogenous processes [5,6,7,8,9,10,11], and the same gene is used there as a treatment-shaping variable: a multi-hit TP53 state is a core variable of the molecular prognostic model (IPSS-M) [12] and redirects therapy, whereas in solid tumors, TP53 status, although it carries prognostic information [13,14], rarely dictates treatment.

Here, we place the two states side by side in a single pipeline covering 29 solid-tumor types (10,639 samples), 19 hematologic malignancy cohorts (10,042 samples) and a 42,714-sample post-therapy clonal-hematopoiesis (CHIP) cohort derived from patients with non-hematologic cancers, for a total of approximately 64,000 samples, and ask three questions in turn. Which state is selected where, and what input explains the choice? What does the state do—does the retained-allele dose carry consequence, and is the wild-type allele gone? And what does the state offer clinically? The answers converge on a single conclusion: the two TP53 states are separated by measurable differences in class balance, allelic state and outcome, and the therapeutic opportunities differ accordingly.

## 2. Results

### 2.1. Two States of One Gene, and How They Are Measured

Two states, not a continuum, are the subject of this analysis: a truncating mutation that removes the protein, and a missense (or in-frame) mutation that leaves a full-length product. Mutation class alone cannot establish whether a full-length mutant protein actually persists, because the wild-type allele may be lost in addition to the mutation. We, therefore, integrated discrete copy-number calls at the TP53 locus for every TP53-mutant sample in which they were available and classified allelic state into four categories: truncating mutation with copy loss (biallelic inactivation), truncating mutation with neutral copy number (monoallelic truncation), missense or in-frame mutation with copy loss (mutant-only, wild-type allele lost), and missense with neutral copy number (heterozygous mutant) (Figure 2, Supplementary Table S1). Throughout, “retained” refers to the mutation-class expectation of a full-length product; the allelic-state analysis in Section 2.3 establishes where that expectation can be confirmed by copy number.

To test whether this rule inflates the compartment difference, we re-analyzed every sample under two alternative rules. Counting a sample as truncating whenever any truncating variant was present gave 34.3% in solid and 24.6% in hematologic samples (odds ratio 1.60, 95% CI 1.34–1.89), essentially identical to the severity-ranked rule (34.2% and 24.4%; odds ratio 1.61, 1.35–1.91); counting a sample as truncating only when every nonsilent variant was truncating narrowed both values but widened the gap (30.4% versus 16.0%; odds ratio 2.29, 1.88–2.79). Compound samples carrying both a truncating and a protein-producing variant were 3.9% of solid and 8.7% of hematologic samples, and these are exactly the samples in which a full-length protein is retained despite a truncated allele (Supplementary Table S3).

### 2.2. Which State Is Selected Where, and What Writes the Spectrum?

If the compartments differ in which state their tumors carry, the first signature should be the spectrum itself. The two compartments differ at the level of the allelic state itself: where copy number resolves the state, solid tumors are dominated by loss of the wild-type allele (19.5% biallelic inactivation and a further 39.2% carrying a missense mutation with copy loss, so that 60.6% of missense-mutant solid tumors retain no wild-type copy), whereas hematologic disease is dominated by a retained full-length protein (66.7% heterozygous mutant and only 7.1% biallelic inactivation) (Figure 3). Mutation frequency, the conventional first readout of TP53 in a cohort, differed by more than two orders of magnitude: across the 31 TCGA cancer types that contained a TP53-mutant sample, it ranged from 0.4% (thyroid carcinoma, 2/500) to 91.2% (uterine carcinosarcoma, 52/57), pooled 35.3% over 10,887 samples, and across the hematologic tumor cohorts, it ranged from 2.0% to 61.9%, pooled 10.9% over the 18 lineage-defined cohorts (7659 samples) (Figure 4a and Figure 5a). Because that axis is set by entity and by sampling design rather than by the state of the mutation, the comparisons below use mutation class and allelic state rather than rates. Classifying each TP53-mutant sample by its most severe alteration, truncating mutations (nonsense, frameshift, splice-site) accounted for 34.2% of solid-tumor alterations but only 24.4% of hematologic ones (χ^2^ = 33.7, df = 3, *p* = 2.3 × 10^−7^), with missense mutations correspondingly more dominant in blood (Figure 4b and Figure 5b). The difference is modest in absolute terms (9.8 percentage points; odds ratio for truncation 1.61, 95% CI 1.35–1.91; Cramér’s V = 0.085), so the argument developed here rests on the direction and reproducibility of the difference rather than on its magnitude.

It was not a detection artifact. Restricted to whole-exome/whole-genome cohorts, truncating mutations remained more frequent in solid tumors (34.2%) than in hematologic disease (23.6%; χ^2^ = 9.9, *p* = 1.7 × 10^−3^). The difference also survived patient-level deduplication (34.3% of 3814 patients versus 24.6% of 1084 patients; odds ratio 1.60, 95% CI 1.37–1.86). Finally, it was reproduced in the NCI TP53 database (release 21, the successor to the IARC database), where truncating mutations were again more frequent in solid than hematologic disease across 25,978 samples (22.1% vs 16.1%; χ^2^ = 32.8, *p* = 1.0 × 10^−8^; the lower absolute fractions reflect that database’s predominantly Sanger-based composition) (Supplementary Figures S1 and S2a). That comparison draws on material of mixed provenance in both arms and more so on the hematologic side (cell lines 18.2% versus 6.5% of entries); excluding every cell-line entry from the database preserves and strengthens the result (21.7% versus 13.8%; odds ratio 1.74, 95% CI 1.48–2.03; *p* = 7.3 × 10^−12^).

Platform composition does not explain the difference either. In the platform-matched set used above (hematologic cohorts assayed by whole-exome or whole-genome sequencing, *n* = 220), the truncating fraction is 23.6%; widening that group to every hematologic cohort assayed by whole-exome or whole-genome sequencing (*n* = 686, which adds the MDS and B-NOS cohorts) gives 23.5%, and the targeted-panel cohorts give 28.8% (*n* = 146). The panel-versus-whole-exome contrast is not significant (χ^2^ = 1.83, *p* = 0.176) and runs opposite to the direction expected if panels were missing truncating variants, while the whole-exome hematologic comparison with solid tumors remains significant (χ^2^ = 30.25, *p* = 3.8 × 10^−8^; Supplementary Table S3). Individual cohorts nevertheless span a wide range (14.5% in the B-NOS cohort to 76.9% in the smallest CLL cohort), and several cohort-level cells are too small to support inference.

A third resource, assembled from a different body of publications and annotated independently of both of the above, reproduces the asymmetry at larger scale. We re-derived the class balance from the COSMIC Mutant Census (release v104, GRCh38), assigning every sample carrying a TP53 record to a compartment through COSMIC’s own phenotype classification and retaining all TCGA-derived samples: 48,085 classifiable solid-tumor samples and 3258 hematopoietic and lymphoid samples. Truncating mutations accounted for 30.0% of solid and 20.4% of hematologic samples (odds ratio 1.68, 95% CI 1.54–1.83; three-class χ^2^ = 136.9, df = 2, *p* = 1.9 × 10^−30^) (Supplementary Figure S3a and Table S2). The direction, therefore, agrees with our own pipeline, and the effect size is closely similar (odds ratio 1.61), in a resource whose hematologic arm is larger than ours and whose samples were classified from COSMIC’s annotations rather than from ours. The contrast did not depend on the material analyzed or the assay used: excluding cell lines gave 30.0% versus 20.5%, restriction to primary lesions 29.2% versus 20.5%, whole-exome samples 32.0% versus 20.2%, panel-sequenced samples 28.0% versus 20.4%, and patient-level deduplication 29.5% versus 20.5%; removing each solid site in turn left the odds ratio between 1.57 and 1.76 across 43 refits. Across individual sites, the truncating fraction ranged from 19.1% to 40.8% among the 20 solid sites with at least 200 samples, and only 1 of them—the central nervous system, at 19.1%—fell below the hematologic pooled value of 20.4% (Supplementary Figure S3b). COSMIC’s coverage marks the boundary of this replication, and it is the same boundary that limits the allelic-state analysis: 17.3% of its TP53 records carry no consequence annotation, and zygosity or loss-of-heterozygosity values are present for 13.0% and 0.7% of them, so this resource corroborates the mutation-class contrast but cannot resolve which allele a missense mutation leaves behind.

Because pooled samples are not independent observations, we repeated the comparison with each cohort as a single unit: the median truncating fraction was 36.0% across 20 solid cohorts versus 23.4% across 20 hematologic cohorts (Mann–Whitney *p* = 0.029). The direction, therefore, survives a test that sample-level pseudoreplication cannot inflate, but the hematologic distribution is wide (per-cohort truncating fractions 0–76.9%), a heterogeneity addressed in the limitations. The same test applied within COSMIC, where studies are identified explicitly, gave median truncating fractions of 28.1% across 55 solid studies versus 15.0% across 6 hematologic studies; 6 hematologic studies leave that comparison underpowered (*p* = 0.38), so the cohort-level check rests on our own pipeline rather than on the replication.

Restricting the same NCI database to whole-genome/whole-exome entries reverses the direction (solid 31.2%, *n* = 913 versus hematologic 48.8%, *n* = 43; *p* = 0.024; Supplementary Figure S2b). We examined that subset directly rather than setting it aside. The discrepancy reflects its sampling frame rather than the sequencing platform: all 43 hematologic entries are established cell lines (43/43), whereas the solid arm is predominantly patient-derived surgical specimens (430 of 913 surgical; 205 cell lines, 14 xenografts, 264 unspecified). Cultured cell lines are selected for in vitro growth and are enriched for entities with high truncating fractions; they are not a patient cohort. Removing the largest entities from the subset did not restore the patient-based direction (48.6–51.4% across the remaining 35–38 samples), confirming that the composition of the subset, not the platform filter, drives the reversal. The patient-based platform-matched comparison is accordingly the one performed in our own pipeline (Figure S1), in which the direction is preserved: 34.2% of 3818 TCGA exomes versus 23.6% of 220 whole-exome/whole-genome hematologic samples (*p* = 1.7 × 10^−3^).

Within hematologic entities, the class balance is graded rather than uniform. De novo AML (TCGA) is 50% truncating, whereas pediatric AML is 11%, indicating that when the context favors complete inactivation—often through 17p deletion together with a multi-hit, biallelic mutational state [12]—the loss-of-function profile reappears.

The composition difference could reflect different mutational inputs rather than different meanings, and, here, the two compartments genuinely part company, because what writes the spectrum differs. Because deamination of 5-methylcytosine at CpG dinucleotides is the dominant endogenous source of TP53 missense mutations, we used the fraction of arginine-residue missense mutations (R→H/Q/C) as a proxy for this process. Solid and hematologic cancers gave nearly identical values: 20.7% of 2,717 solid missense mutations versus 21.2% of 780 hematologic ones—a difference of −0.4 percentage points (95% CI −3.7 to +2.8; *p* = 0.83). Because a non-significant test does not by itself establish equivalence, we tested equivalence directly: the confidence interval lies wholly within a pre-specified ±5 percentage-point margin, which supports equivalence at that margin but not at a stricter ±3 percentage-point margin. This similarity is expected if exogenous mutagens account for most of the between-cancer variation in the solid spectrum [5,6]: in their absence, both compartments settle on the same endogenous baseline. Whole-genome analyses of treatment-naive hematologic entities support this reading, reporting predominantly endogenous processes: 5-methylcytosine deamination and APOBEC in pediatric AML [7], core-binding-factor AML [8], and B-precursor ALL [9], activation-induced cytidine deaminase in CLL [10], and little APOBEC activity in DLBCL [11]. The between-solid spread of the proxy (0–38%) (Supplementary Figure S4a) reflects that exogenous overlay, whereas the narrower within-hematologic spread (Supplementary Figure S5a) (40.5% in BeatAML AML, 8.3% in myeloma, 0% in one T/NK cohort) tracks proliferative and repair context; the proxy was not associated with patient age (*p* = 0.57), consistent with an endogenous (replication/repair) rather than age-accumulated origin.

Restricting the analysis to missense mutations separates the two components of this comparison. The CpG-transition fraction among missense mutations did not differ detectably between compartments (563/2717, 20.7%, versus 165/780, 21.2%; χ^2^ = 0.07, *p* = 0.79), whereas hotspot occupancy did: 32.4% of solid and 29.7% of hematologic missense mutations fall in the six canonical hotspots, and most CpG-type mutations are themselves hotspot mutations (502 of 563 solid, 143 of 165 hematologic). The compartments, therefore, share an endogenous baseline and differ in hotspot architecture rather than in the CpG process itself (Supplementary Table S3).

Hotspot architecture tells the same story from a third direction. Hotspot heterogeneity across solid types was extreme (χ^2^ = 618.6, *p* = 7.5 × 10^−72^), with aflatoxin-driven R249S and UV tandem transitions as canonical exposure fingerprints (Supplementary Figure S4b). In blood, hotspots were less concentrated but remained non-random: pooled across all cohorts, no single hotspot exceeded 12% of mutant samples, yet within an entity, the concentration can be much higher, and each entity showed its own pattern. MDS concentrated on the DNA-contact residue R273H, pediatric AML on R248Q (27% of the mutant samples in that cohort), CLL favored the conformational class (R175H its top hotspot), and multiple myeloma showed little concentration (Supplementary Figure S5b). Because hotspots concentrate within the DNA-binding domain (Supplementary Figure S4c), and specific residues correspond to distinct structural classes of mutant p53, this entity-restricted architecture in blood is more consistent with selection on the properties of the retained mutant protein than with any exogenous mutagen.

Three independent spectrum-level readouts (class balance, endogenous baseline, and hotspot geometry), therefore, point the same way. In solid tumors, the spectrum is written from outside: exogenous mutagens imprint it, and the gene is frequently discarded. In blood, the spectrum is written from within: endogenous processes supply the mutations, and selection on the retained protein shapes which of them persist.

### 2.3. What the State Does: Retained-Allele Dose and Loss of the Wild-Type Allele

A spectrum-level contrast implies, but does not by itself prove, a functional contrast. The functional test is the retained-allele dose. If hematologic selection acts on the missense protein, patients retaining more mutant alleles should fare worse. In the MDS IPSS-M cohort (*n* = 3173), patients with ≥2 TP53 mutations (a sequence-only proxy for biallelic, multi-hit state [12]) had worse survival than those with a single mutation (hazard ratio (HR) 1.78; P = 1.0 × 10^−4^). A caveat attaches to this contrast: multi-hit TP53 status is itself an entry variable of IPSS-M, so the gradient documented here overlaps with that model and is not independent evidence for it; what it shows is that the number of retained mutant alleles is associated with outcome, not that it adds prognostic information beyond the established score. Because no mutant protein was quantified in these cohorts, the association is not evidence of increased mutant-p53 dosage, and a multivariable Cox model adjusting for age confirmed an allele-count association: multi-hit conferred a 4.05-fold hazard (95% CI 3.25–5.05) versus 2.12-fold for single-hit (Figure 6a, Supplementary Figure S6). In the two AML cohorts, the same gradient could not be demonstrated rather than being contradicted: only 4 of 186 patients (TCGA) and 16 of 497 patients (BeatAML) had multi-hit disease, and multi-hit status was not associated with worse survival (hazard ratios 1.03, 95% CI 0.29–3.61, and 0.77, 95% CI 0.40–1.48), with intervals far too wide to support or exclude an allelic-dose effect. TP53 mutation itself was nevertheless strongly adverse in both AML cohorts (mutant versus wild-type hazard ratios 7.66, 95% CI 3.13–18.75, and 12.92, 95% CI 7.11–23.47) [15,16]. The graded retention effect is, therefore, established in the large MDS cohort; whether it operates in AML remains unresolved.

Because multi-hit status is one of the variables from which IPSS-M is built, the gradient reported here cannot be read as independent prognostic evidence: it partially reconstructs a score that already contains the same variable. We, therefore, use it only for what it can support—that the amount of retained mutant sequence tracks outcome—and not as an estimate of added prognostic value beyond the established model.

The gradient is the functional counterpart of the composition difference. In a compartment that tolerates truncation, the operative event is loss, and the number of retained mutant alleles should be irrelevant; in a compartment that retains the protein, the retained-allele load is what selection could act on, and in MDS, it behaves accordingly. Because mutation class is assigned from sequence alone, “retention” as used here denotes the mutation-class expectation of a full-length product rather than a directly measured allelic state; this section integrates copy-number calls where they exist, which resolves allelic state in the solid arm but not in the hematologic arm. The dose effect in MDS is, therefore, a prediction of the retention model that is consistent with the data rather than a measurement of retained protein, and its extension to hematologic disease generally remains untested.

In the solid arm, copy-number data were available for all 3748 TP53-mutant TCGA samples (Figure 2a). Biallelic inactivation (a truncating mutation together with loss at the locus) accounted for 19.5% of TP53-mutant tumors, and a further 39.2% carried a missense mutation with copy loss at the same locus, so that 60.6% of missense-mutant solid tumors had lost the wild-type allele and retained only the mutant. Only 25.4% of TP53-mutant solid tumors were simple heterozygous missense. The solid compartment is, therefore, characterized not merely by tolerance of truncation but by loss of the wild-type allele as the operative event; whichever class the mutation belongs to, the wild-type copy is usually gone.

Two points follow, and both matter for how the two-state framing should be read. First, the most common allelic configuration in solid tumors—a missense mutation with loss of the wild-type allele, 39.2% of samples with copy-number data—is a “protein-present” state, not a protein-loss state: the wild-type allele is gone, but a full-length mutant protein is still produced, and dominant-negative or gain-of-function activity can persist in exactly this configuration. Second, the two-state axis used throughout refers to the presence or absence of a full-length protein; it is not a statement about allelic completeness, which is why loss of heterozygosity does not move a sample into the loss state. The four states, what each implies for protein presence and for the wild-type allele, and their frequencies in the two compartments are tabulated in Supplementary Table S4.

In the hematologic arm, the same analysis could not be completed at usable scale, and we report it as unresolved rather than resolved. Only 420 of 1187 TP53-mutant hematologic samples (35%) had copy-number calls (Figure 2b), and 378 of those came from a targeted panel (MSK-IMPACT), which detects homozygous deletions but not shallow losses; among those samples, biallelic inactivation was 2.5%. The remaining 42 samples came from array-based GISTIC studies (TCGA de novo AML and DLBCL), where biallelic inactivation was 35.7% (34.4% in AML alone), the entity that our own class analysis already identifies as the outlier with the highest truncating fraction. Because the two platform strata point in opposite directions, hematologic allelic fractions are reported stratified by platform and are not used for compartment-level inference. The consequence for interpretation is explicit: the mutation-class contrast in blood (Section 2.2) stands, but the retention model it motivates remains a prediction from mutation class rather than a measured allelic state, pending copy-number-resolved hematologic cohorts.

The solid arm, where copy-number resolution is complete, allows for the converse question: does the way p53 function is lost matter for outcome among TP53-mutant patients? We linked the allelic-state classification to overall survival for 3684 TP53-mutant solid-tumor samples with available follow-up across the 29 solid-tumor types and fitted, within each tumor type, a Cox model contrasting each state against retained missense-only (heterozygous missense, the state that keeps a full-length protein without further loss), pooling the log hazard ratios across types. The answer was negative in every direction: biallelic inactivation versus retained missense-only gave a pooled hazard ratio of 1.04 (95% CI 0.86–1.26), monoallelic truncation 0.98 (0.80–1.21), and mutant-only with copy loss 1.09 (0.93–1.27), with negligible between-type heterogeneity (I^2^ = 0–35%) (Figure 6b). The cohort statistics reported here are those of the 3657 patients who carried one of the four allelic states and had a positive recorded survival time: 1391 deaths (38.0%), with a median follow-up of 24.0 months among censored patients (this is slightly smaller than the 3684 samples entered into the per-type models because samples without a positive survival time were excluded). Because the proportional-hazards assumption was not met within the largest tumor types (state × log-time interaction *p* < 0.05 in all six), the contrast was re-estimated with a proportional-hazards-free (Peto/log-rank) estimator, which gave the same conclusion: pooled hazard ratios 0.93 (0.77–1.13, *P* = 0.48), 0.98 (0.80–1.20, *p* = 0.83) and 0.89 (0.77–1.04, *p* = 0.14) for the three contrasts; Benjamini–Hochberg adjustment leaves all three non-significant. The null result is, therefore, not an artefact of the modeling assumption, although it remains unadjusted for stage and treatment. Among TP53-mutant solid tumors, how p53 function is lost carries no measurable survival signal.

Adjusting for the strongest available confounders does not change that answer. Adding sex and AJCC pathological stage to the age-adjusted Cox model gave pooled hazard ratios of 1.06 (95% CI 0.87–1.28) for biallelic inactivation and 1.02 (0.88–1.18) for mutant-only with copy loss, and adding history of neoadjuvant therapy gave 1.05 (0.89–1.25) and 1.02 (0.88–1.18); restricting the analysis to the 2,448 patients with a recorded stage gave 1.08 (0.86–1.35) and 0.99 (0.82–1.19). Because the proportional-hazards assumption was violated in the largest tumor types, we describe the result as no difference detected rather than as equivalence, and we retain the Peto and stratified analyses as robustness checks. Exposure to specific treatments is not captured in these public cohorts, which remains a limitation.

Figure 6 places the two compartments side by side: the hematologic panel shows an allele-count gradient that reaches significance in the large MDS cohort, while no solid-tumor state separates from the others. The contrast with the hematologic side is, therefore, state-specific rather than universal. In blood, the retained-allele count is graded against outcome, in the direction a protein-selection model predicts; because no protein was measured, this is consistent with, but not direct evidence for, a dosage effect; in solid tumors, once p53 function is gone—whichever way—no survival difference was detected, consistent with loss being tolerated because it is achieved cheaply and early. The two states of TP53, thus, differ not only in how often each compartment selects them but in whether the state carries prognostic weight at all: it does where the protein is retained and targeted by selection, and it does not where the protein is dispensable.

### 2.4. What the State Offers: A Target Only in the Retained-Protein Compartment

The clinical meaning of a TP53 mutation begins with its frequency, and even that reads differently across sampling designs, a warning against naive cross-cohort comparison. Within the same entities, sampling design moved the apparent mutation rate substantially: CLL was 7.9% in a population-anchored cohort (*n* = 1154) versus 61.9% in a clinically enriched referral cohort (*n* = 21); DLBCL was 10.4% (TCGA) versus 23.0% in a referral cohort. Pooling naive and clinically enriched cohorts without stratification (9.0% versus 18.0% pooled) contaminates cross-study comparisons (Supplementary Figure S5c).

What therapy then does to the TP53-mutant clone is the sharpest clinical question the two states pose. We re-analyzed a 42,714-sample post-therapy CHIP cohort derived from patients with non-hematologic cancers: 1.2% (493 samples) carried a TP53-mutant clone, and missense mutations dominated (89% of mutated samples), with Y220C the most frequent hotspot. This is the reservoir from which therapy-related myeloid neoplasms emerge [17,18]. Paired tumor–blood cohorts independently confirm that TP53 and PPM1D mutations are enriched in patients with prior chemotherapy [19], and lineage tracing shows such clones pre-exist chemotherapy and are selected rather than induced [20]; the cross-sectional design of the present analysis cannot by itself distinguish selection from induction, and we, therefore, treat the temporal model as one supported by those studies rather than demonstrated here.

In blood, TP53 mutation, therefore, behaves as a treatment-defining variable: it anchors molecular prognostic scoring [12], its retained-allele dose—a sequence-based proxy, not a measured protein quantity—tracks outcome where the gradient is measurable (Section 2.3), multi-hit disease is not rescued by allogeneic transplantation [15], and the post-therapy reservoir is already missense-dominant and hotspot-organized before any selective pressure of the next treatment is applied. In solid tumors, the mutation instead enters clinical decision making as one factor among many: same gene, different states, and different clinical weight.

## 3. Discussion

The findings support a single thesis: TP53 mutation exists in two states—protein lost or protein retained—and the compartments differ in which state their tumors carry and in what that state is worth. We are not aware of a previous quantitative side-by-side comparison of the two states across compartments, although each state has been studied extensively within its own compartment. Etiologically, the solid-tumor spectrum is an exposure record written by ultraviolet light, tobacco smoke, aflatoxin, and other exogenous mutagens, as six decades of molecular epidemiology established [2,3,4], whereas the hematologic spectrum is written by endogenous processes and organized by selection, as the equivalent CpG baseline (20.7% versus 21.2%; difference −0.4 pp, 95% CI −3.7 to +2.8) and the entity-restricted hotspot architecture indicate [5,6,7,8,9,10,11]. Functionally, solid tumors tolerate and often favor truncating loss (34.2% versus 24.4%, an odds ratio of 1.61 for truncation in solid disease; reproduced in the NCI TP53 and COSMIC databases and preserved at cohort level), and where copy-number data resolve allelic state, they show loss of the wild-type allele to be the operative event in that compartment (19.5% biallelic inactivation and copy loss in 60.6% of missense-mutant tumors), whereas hematologic disease retains full-length missense protein by mutation class [1], with allelic state unresolved at usable scale. In the large MDS cohort, outcome tracks the retained-allele dose (multi-hit versus single mutation: hazard ratio 1.78; multivariable 4.05-fold versus 2.12-fold) [12,15,16]. Clinically, the mutation is a modifier in one compartment and a treatment-defining variable in the other, most starkly in the post-therapy CHIP reservoir, which is already missense-dominant (89%) and Y220C-led before the next treatment acts [17,18,19,20]. The clinical contrast drawn here is one of role rather than of prognostic strength: among TP53-mutant solid tumors, the allelic state carried no measurable survival signal (Section 2.3; Figure 6b), and the comparison rests on where the mutation enters decision making in each compartment.

Why the two compartments select for different TP53 states remains an open mechanistic question, and the data here suggest candidates rather than answers. A truncating change removes p53; a missense change yields a dominant-negative and gain-of-function protein that, unlike simple loss, also interferes with the p53-family members p63 and p73 and acquires pro-survival, metabolic, and invasive activities [1]. Hematopoietic lineages are among the tissues most prone to p53-family-dependent apoptosis after genotoxic stress and are the direct targets of cytotoxic therapy, so a retained full-length mutant protein that blocks p53-family-dependent death while acquiring oncogenic functions may confer advantages that loss cannot. This is a candidate explanation for missense retention in blood, and it is testable by functional comparison across compartments. Conversely, in solid tumors, where chromosomal instability makes arm-level loss routine, biallelic inactivation is achievable at low cost and was observed here in roughly one-in-five TP53-mutant tumors, with the wild-type allele lost in most missense-mutant cases as well. Whether lineage-specific apoptotic wiring, differential tolerance of aneuploidy, or the tissue-restricted mutational processes documented above dominate this divergence is a question for experimental models.

This reframing repositions full-length mutant p53 as the shared currency of hematologic TP53 disease and, therefore, as the natural target. The retained protein is the substrate on which allele-specific strategies act: rezatapopt, a reactivator selective for the Y220C mutant, restores wild-type conformation in Y220C-mutant cells [21,22,23], although in AML, this is incompletely translated into apoptosis [24]; TCR-based reagents recognize p53-mutant peptide–HLA complexes such as R175H presented on HLA-A*02:01 [25,26,27]. Such reagents can act because p53-derived peptides reach the cell surface: although p53 is a nuclear protein, mutant p53 is degraded by the proteasome and its peptides are presented on MHC class I [26], so a retained mutant protein remains visible to T-cell-based reagents even after loss of the wild-type allele. The missense dominance documented here (including Y220C as the top hotspot of the post-therapy reservoir) is the quantitative basis for these allele-defined programs, and the MDS dose gradient indicates that the retained-allele load is biologically consequential rather than a passive marker. We note that this is a perspective drawn from mutation spectra, not a comparison of treatment outcomes: the present study contains no clinical intervention data, rezatapopt and related reactivators are in development across both solid and hematologic tumors, and the positioning of allele-specific strategies in blood is a hypothesis for prospective testing rather than a conclusion of this analysis.

The two states also address an apparent paradox in the therapy literature. The prevailing narrative, that chemotherapy causes TP53-mutant leukemia and MDS, is difficult to reconcile with spectra that are already missense-dominant and Y220C-led at diagnosis and in the pre-therapy reservoir. The cited paired and lineage-tracing studies support a temporal model in which the clone exists before therapy and therapy edits it: cytotoxic treatment selects pre-existing TP53-mutant clones [17,18,19,20], converting a molecular-reservoir event into clinical prominence. Our own analysis is cross-sectional and cannot independently distinguish selection from induction, so we present this model as one supported by those studies. Loss of function and retention are not opposite mistakes but two sides of the same selective logic. The phase-2/3 experience with eprenetapopt, magrolimab, and other agents [28,29,30], together with the contrasting success of non-genotoxic, allele-defined approaches in CLL [31,32,33], indicates that the design principle of targeting the retained full-length protein with non-genotoxic, allele-specific tools is among the most promising directions for the field on current evidence, and that it applies where the retained-protein meaning applies: in blood.

### Testable Predictions

1. In solid tumors, strategies that restore the wild-type protein should have little to act on, because the wild-type allele is usually lost alongside the mutation; loss of that allele does not, however, remove the mutant protein as a target. Most TP53-mutant solid tumors, therefore, still carry a full-length mutant protein that mutant-selective reactivators and peptide–HLA reagents could in principle address, even though restoration strategies that depend on a remaining wild-type allele are confined to the minority of tumors that keep a full-length protein heterozygous.

2. In hematologic disease, the retained-allele count should predict response to protein-directed agents: if the mutant protein is the target, then tumors with more retained mutant alleles (multi-hit, or mutant-only with copy loss) should benefit more from conformational reactivators and mutant-peptide reagents than single-hit disease. What the MDS gradient documents is an association between allele count and outcome at the sequence level, not measured mutant protein abundance; the quantity to test prospectively is, therefore, allele dosage as a sequence-derived expectation, together with the direct protein measurements that these public cohorts do not provide.

3. Therapy-related myeloid neoplasms should arise preferentially from the protein-retaining state: the post-therapy reservoir reported here is already missense-dominant and Y220C-led before any new selective pressure, which predicts that the pre-treatment genotype of the clone—not the treatment alone—determines which patients are at risk.

Limitations are those of public-data meta-analysis, and several are material. Cohorts differ in platform, depth, and clinical enrichment; the hematologic compartment is markedly heterogeneous (per-cohort truncating fractions 0–76.9%), so compartment-level statements describe distributions rather than a uniform biology. Allelic state could be integrated only where public copy-number calls exist: this was complete for the solid arm (all 3748 samples, GISTIC) but covered just 35% of hematologic samples, predominantly from a targeted panel insensitive to shallow loss. The two platform strata disagreed (2.5% versus 34–40% biallelic inactivation), so “retention” in blood denotes a class-based expectation rather than a measured allelic configuration, and the allelic-dose effect is established only in MDS. Copy-neutral loss of heterozygosity and variant allele fractions were not captured, and the independent COSMIC replication cannot supply them either, since zygosity and loss-of-heterozygosity fields are populated for 13.0% and 0.7% of its TP53 records; that resource, therefore, corroborates the mutation-class contrast but leaves the allelic state unresolved on both sides of the comparison. The CpG proxy, although equivalent across compartments within a ±5-percentage-point margin, is a coarse stand-in for signature-level analysis. The marginal *p* values of exploratory comparisons (for example, the NCI cell-line subset, *p* = 0.024) were not corrected for multiplicity. The deeper caution is conceptual: pan-cancer narratives that treat TP53 mutation as a single entity average away precisely the compartment conditionality documented here. Finally, germline TP53 status (for example, Li-Fraumeni spectrum) was not captured by the public tumor-only datasets and is outside the scope of this somatic analysis.

Two further boundaries deserve statement. The two states are a deliberate simplification along one axis—presence of a full-length protein—and not a complete taxonomy: finer partitions, such as transitions at CpG versus non-CpG sites, or the structural classes of missense mutants are not modelled here and would partition the same samples differently. Ancestry and population categories are not available in these cohorts, so no population-level inference is offered, and any such comparison would require cohorts with harmonized genetic-ancestry annotation.

## 4. Materials and Methods

**Data sources.** Mutation data were retrieved from the cBioPortal public API. Solid tumors: 32 TCGA PanCancer Atlas studies were retrieved; 31 contained at least one TP53-mutant sample (all except uveal melanoma, for which no TP53 variant is recorded), of which 29 are solid (10,639 samples); the two hematologic types (acute myeloid leukemia, DLBCL) were assigned to the hematologic compartment. Hematologic malignancies: 19 cohorts (10,042 samples) spanning myeloid, B-cell, T/NK-cell, and B/T-precursor lineages, plus the 42,714-sample post-therapy CHIP cohort (20 studies, 52,756 samples in total; study identifiers in the retrieval manifest (results/retrieval_manifest.csv)). Mixed-lineage and CHIP panels were excluded from the core solid-versus-hematologic comparisons.

**Mutation classification.** Nonsilent TP53 mutations were classified as missense, truncating (nonsense, frameshift, splice-site), in-frame indel, or other. Each sample was assigned one dominant class by severity (truncating > in-frame indel > missense > other) to avoid double-counting biallelic samples; silent mutations were excluded.

Metrics. Per cohort, we computed nonsilent mutation frequency, dominant-class composition (cohorts with ≥15 classified mutant samples), hotspot concentration (top-15 missense changes by total count; cohorts with ≥10 missense samples), DNA-binding-domain enrichment (missense at residues 94–312), and the CpG-transition proxy (fraction of missense at arginine residues with H/Q/C substitution). The CHIP cohort was summarized for rate, class, and top hotspot. The CpG proxy is coarse by construction: it detects CpG-associated deamination only through mutations at arginine codons, it is not a signature decomposition, and it, therefore, supports a comparison between compartments rather than an assignment of mutational processes to either.

**Statistics.** Compartmental composition was tested at sample level (χ^2^, with odds ratio and 95% confidence interval) and at cohort level, treating each cohort as one observation (Mann–Whitney U), so that the result does not depend on sample-level pseudoreplication; patient-level deduplication (one dominant class per patient, by the severity rule above) was applied as a sensitivity analysis. Equivalence of the CpG proxy between compartments was assessed from the confidence interval of the difference against a pre-specified ±5-percentage-point margin. Hotspot heterogeneity used an χ^2^ test of the hotspot × cancer-type matrix. A platform-matched sensitivity analysis restricted both compartments to whole-exome/whole-genome cohorts. Discrete copy-number calls at the TP53 locus were integrated where publicly available (GISTIC for the TCGA studies; each hematologic study’s discrete CNA profile otherwise), treating −1 and −2 as copy loss to infer allelic state; because the hematologic calls derive from two assay types with different sensitivity to shallow loss, hematologic allelic fractions are reported stratified by assay and are not used for compartment-level inference. Copy-neutral loss of heterozygosity and variant allele fractions were not captured. Survival: Kaplan–Meier curves and log-rank tests; hazard ratios with 95% confidence intervals by the Peto method; a multivariable Cox proportional-hazards model (Breslow partial likelihood) with single-hit, multi-hit, and age-standardized covariates. Solid-tumor outcome by allelic state: overall survival was taken from the same cBioPortal clinical repositories; within each of the 29 tumor types, a Cox model contrasted each allelic state against retained missense-only (adjusting for age where available) and log hazard ratios were pooled across types by inverse-variance weighting. Proportional hazards were checked with a state × log-time interaction term within each of the six largest tumor types; because the assumption was not met, a Peto (log-rank) estimator, which does not assume proportional hazards, was applied to the same contrasts as a sensitivity analysis, and the three pooled contrasts are also reported with Benjamini–Hochberg adjustment. This outcome comparison is exploratory and is not adjusted for stage or treatment, which are unavailable for most of these cohorts. Analyses were performed in Python 3 (scripts 01–49); the pipeline and its retrieval snapshots are shipped with the reproducibility bundle described in the Data Availability Statement.

**Use of generative AI.** GLM (Z.ai) was used at the direction of the authors for language editing and for the development of analysis code and figure production. The authors reviewed and edited all content generated with the assistance of this tool and take full responsibility for the integrity of the work and the accuracy of its data and conclusions. Generative AI tools do not meet MDPI’s authorship criteria and are not listed as authors of this work.

**Independent validation.** The class difference was re-derived in the NCI TP53 database (release 21; https://tp53.cancer.gov/), classifying per-sample dominant class and tumor compartment from morphology, excluding flagged samples. Because the platform-restricted (WGS/WES) subset of that database consists entirely of cell lines on the hematologic side (43/43) and predominantly of patient-derived surgical specimens on the solid side (430/913), that subset is reported for completeness but is not used as a patient-based validation; the patient-based platform-matched comparison is the one performed within our own cBioPortal pipeline. A second independent resource was analyzed in the same way. Every TP53 record in the COSMIC Mutant Census (release v104, GRCh38; 68,719 records covering 62,009 samples) was classified from its Sequence Ontology consequence as truncating (stop-gained, frameshift, splice-acceptor, splice-donor, start-lost), missense, or other; the 17.3% of records without a consequence term were classified from the amino-acid annotation where it allowed (a stop or frameshift as truncating, a single substitution as missense) and otherwise excluded, and each sample received the most severe class it contained. Compartment was taken from COSMIC’s own classification file, linked by COSMIC_PHENOTYPE_ID: primary site haematopoietic_and_lymphoid_tissue was assigned to the hematologic compartment, an unspecified site (NS) was excluded, and every other site was assigned to the solid compartment. Sample type, tumor source, screening platform and individual identifier came from the COSMIC Sample file; TCGA-derived sample names were retained rather than excluded. Truncating fractions were compared by χ^2^ (2 × 2 and 3 × 2) with odds ratios and Wald confidence intervals, and the comparison was repeated excluding cell lines, restricting to primary lesions, stratifying by screening platform (whole-exome versus targeted panel), deduplicating to one sample per individual, removing each solid site in turn (43 refits), and excluding TCGA-barcode sample names. A cohort-level comparison used COSMIC_STUDY_ID, with studies contributing at least five classifiable samples compared by study-level truncating fraction (Mann–Whitney U). The files used (Cosmic_MutantCensus, Cosmic_Sample and Cosmic_Classification, all release v104 GRCh38) were accessed on 24 September 2026 (checksums in ‘results/retrieval_manifest.csv’) and are used under the terms of the COSMIC license with the required citation [34].

## 5. Conclusions

TP53 mutation exists in two states, loss and retention, and the compartments differ in which state their tumors carry. In solid tumors, it is, above all, a record of the mutagens that shaped a cancer, and of a tumor-suppressor function that could be discarded: where copy number resolves allelic state, the wild-type allele is usually lost along with the mutation. In hematologic malignancies, it is a selected, functional, clinically prominent event: written by endogenous processes, expressed by mutation class as a full-length mutant protein whose allelic dose tracks survival in the largest myeloid cohort studied, and concentrated—before therapy ever acts—in the reservoir from which treatment-related disease emerges. Whether the wild-type allele is retained alongside it remains an open question that public copy-number data cannot yet answer. The practical consequence follows directly: where the meaning is the retained protein, the target is the retained protein, which is why allele-specific reactivation and immune strategies are most immediately applicable—though not exclusively so—in blood cancers, a proposition that requires prospective clinical testing. We do not propose preferential use of mutant-p53-directed therapy in hematologic disease; what the data support is that the retained protein is the substrate such therapies require, and whether that substrate translates into clinical benefit remains to be established in prospective trials.

## Figures and Tables

**Figure 1:**
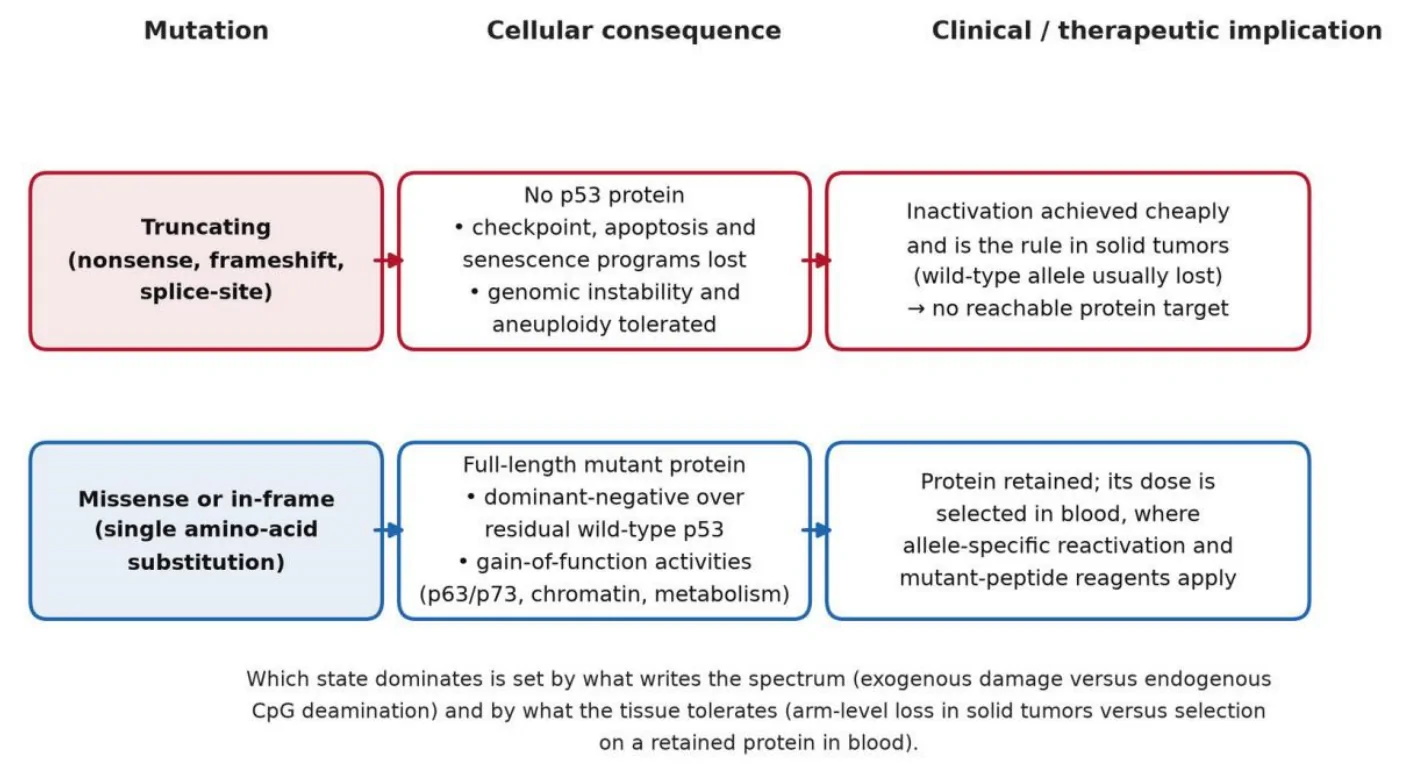
The two states of TP53 mutation. A truncating mutation removes the protein and disables the checkpoint, apoptotic and senescence programs, so that genomic instability and aneuploidy are tolerated; complete inactivation is therefore achieved cheaply and is the rule in solid tumors, where no protein remains to be targeted. A missense mutation instead yields a full-length protein that often retains dominant-negative and gain-of-function activity; that protein is retained in hematologic disease, its dose is selected, and it presents the only reachable target for allele-specific reactivation or mutant-peptide reagents. Which state dominates depends on what writes the spectrum and on what the tissue tolerates.

**Figure 2:**
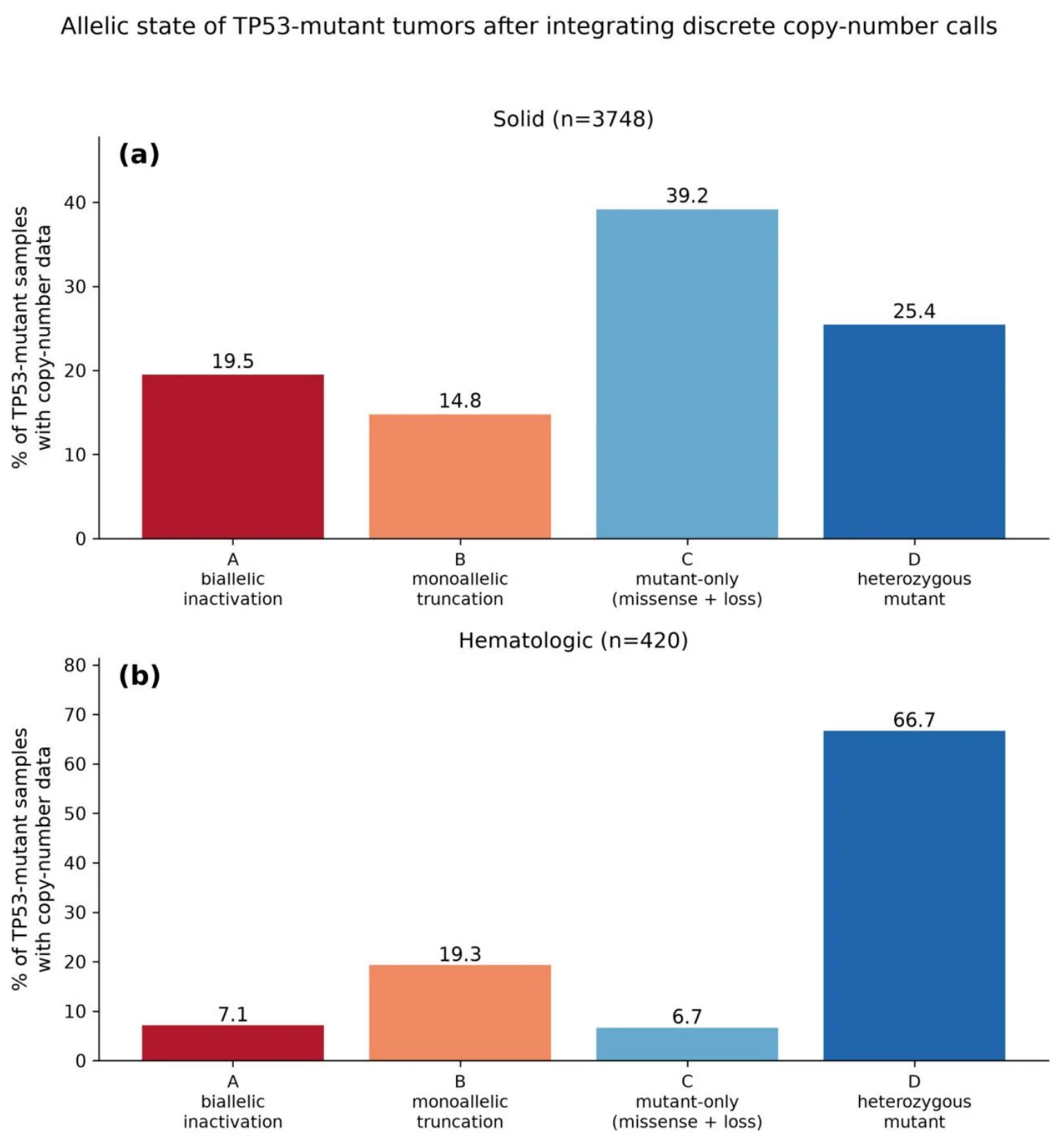
Allelic state of TP53-mutant samples after integrating discrete copy-number calls at the TP53 locus. Categories: biallelic inactivation (truncating mutation plus copy loss), monoallelic truncation (truncating mutation with neutral copy number), mutant-only (missense or in-frame mutation plus copy loss, wild-type allele lost), and heterozygous mutant (missense with neutral copy number). (**a**) Solid tumors (*n* = 3748, all with GISTIC calls) show loss of the wild-type allele as the operative event: 19.5% biallelic inactivation and 60.6% of missense-mutant samples carrying copy loss. (**b**) Hematologic samples are shown for completeness only (*n* = 420 of 1187; 378 from a targeted panel insensitive to shallow loss, 42 from array-based GISTIC studies), and their fractions are platform-dependent; see Supplementary Table S1.

**Figure 3:**
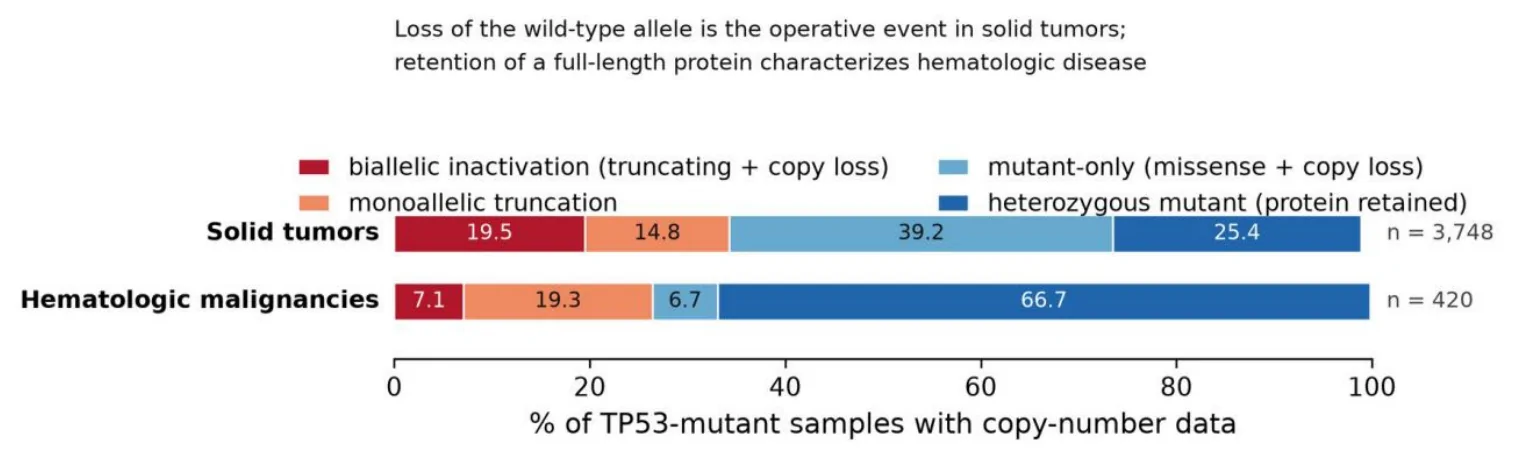
State composition of TP53-mutant tumors in the two compartments. Stacked composition of the four allelic states among TP53-mutant samples with copy-number data: biallelic inactivation (truncating mutation plus copy loss), monoallelic truncation, mutant-only (missense or in-frame mutation with copy loss, wild-type allele lost), and heterozygous mutant (missense with neutral copy number, protein retained). Loss of the wild-type allele dominates the solid compartment, whereas a retained full-length protein dominates hematologic disease.

**Figure 4:**
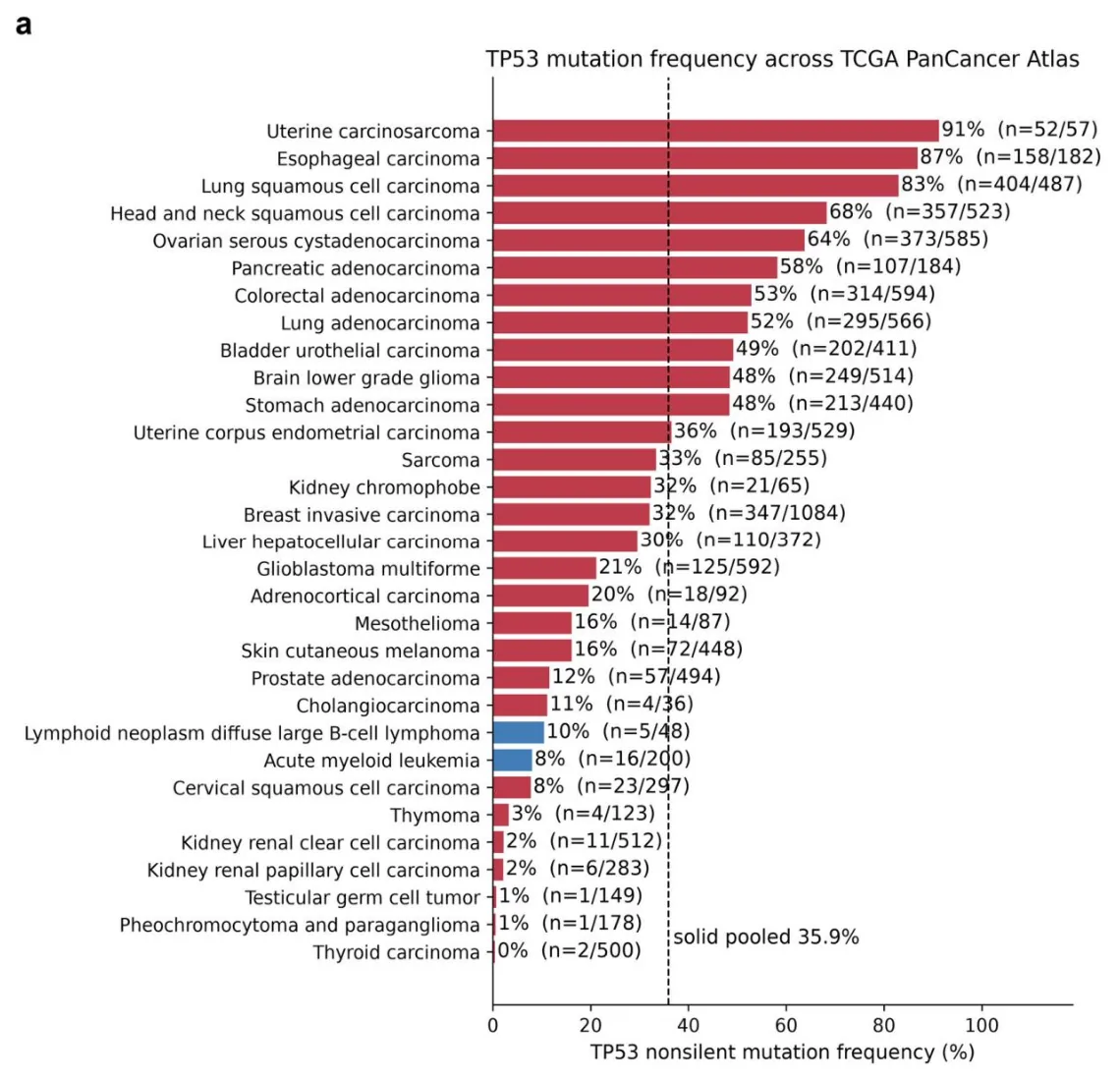

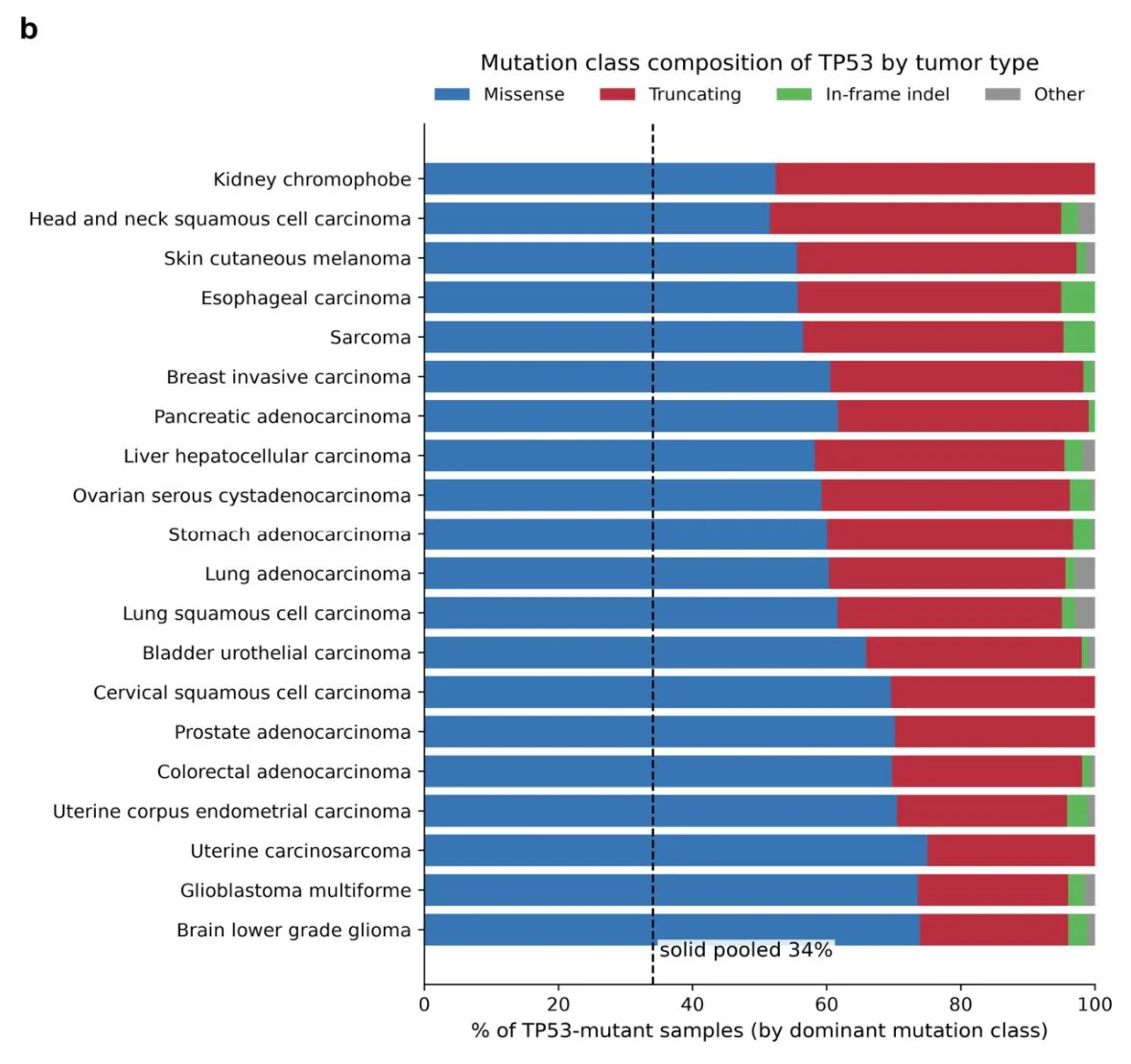
The solid-tumor TP53 spectrum I: mutation frequency and dominant mutation class. (**a**) Nonsilent TP53 mutation frequency across the 31 TCGA PanCancer Atlas cancer types that contained at least one TP53-mutant sample—uveal melanoma, the 32nd study retrieved, contributed none—pooled 35.3%; the two hematologic types are drawn in blue and are assigned to the hematologic compartment, giving a solid-tumor-only pooled frequency of 35.9%. (**b**) Dominant mutation-class composition per solid tumor type (truncating pooled 34.2%; computed from solid types only, *n* = 3818).

**Figure 5:**
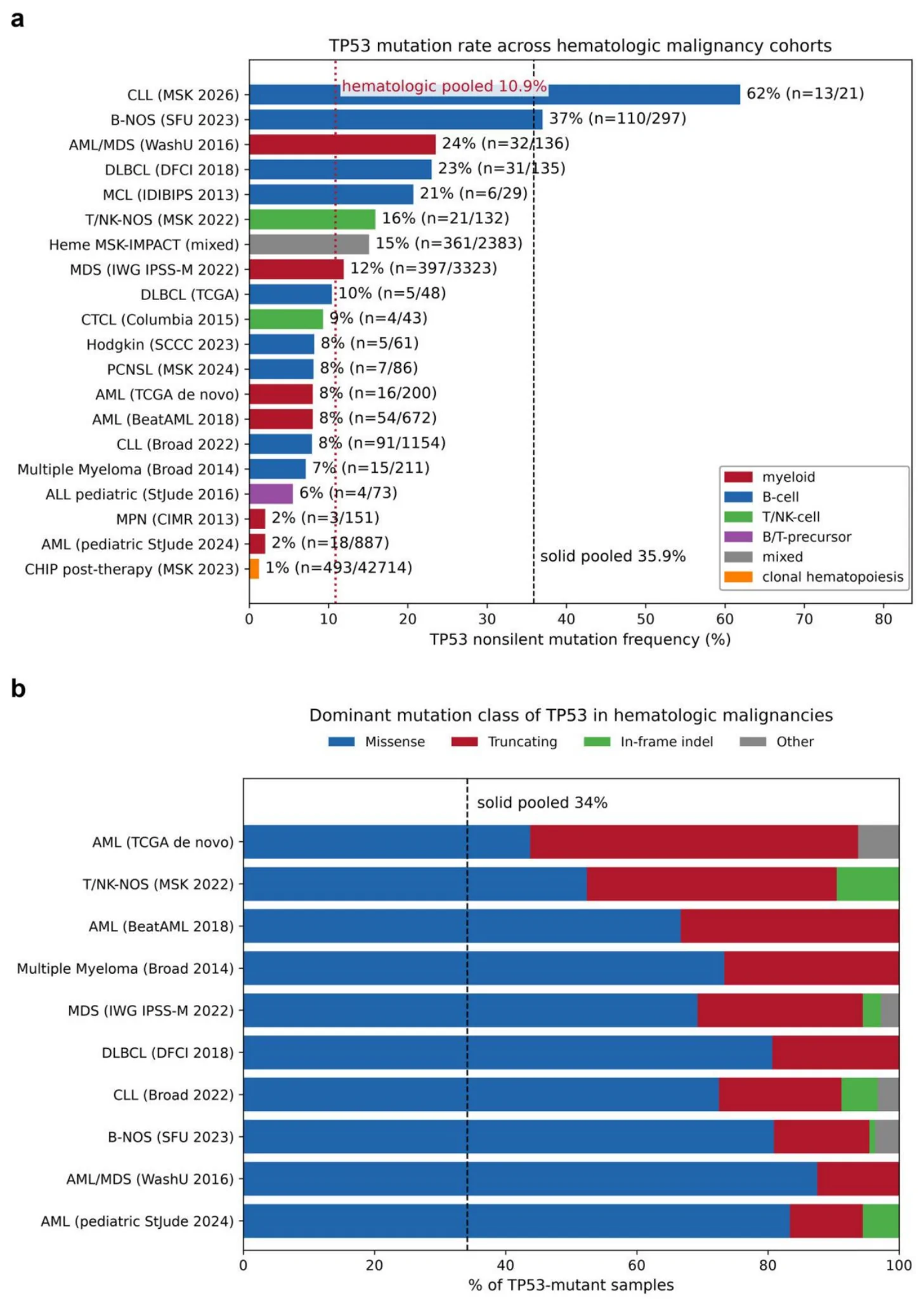
The hematologic TP53 spectrum. (**a**) Nonsilent TP53 mutation frequency across 19 hematologic malignancy cohorts and the post-therapy CHIP cohort, lineage-colored (pooled 10.9% across the 18 lineage-defined cohorts; the CHIP cohort is displayed but excluded from the pooled value). (**b**) Dominant mutation-class composition per cohort (truncating pooled 24.4%; the solid-tumor pooled value of 34.2% is drawn for reference).

**Figure 6:**
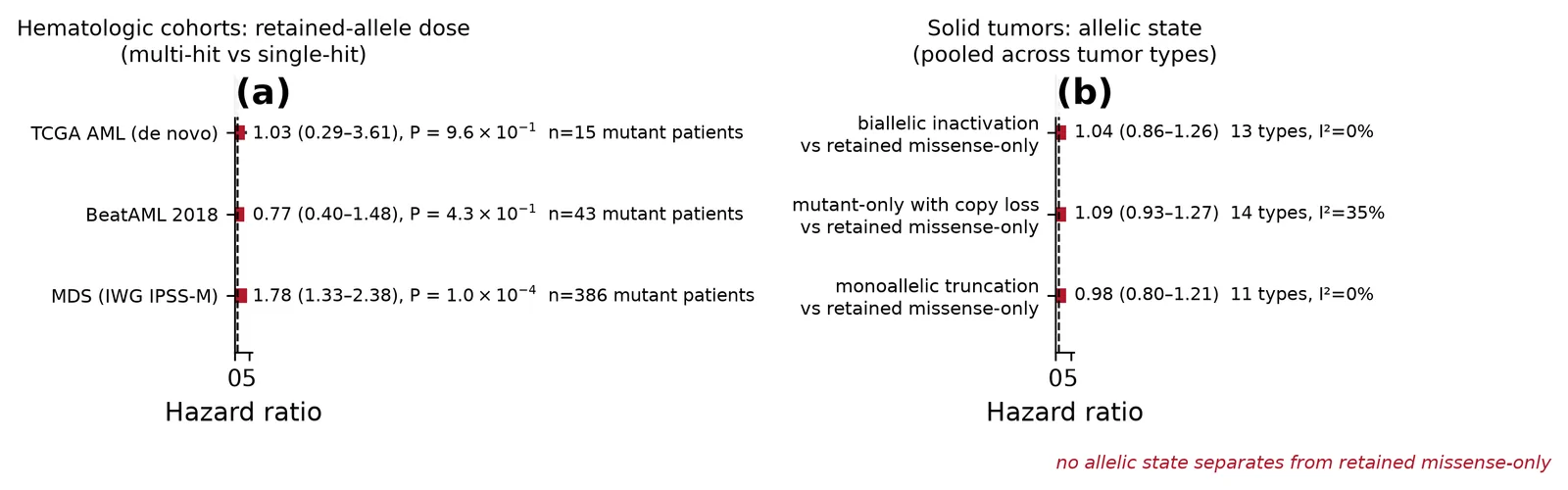
Outcome by TP53 state in the two compartments. (**a**) Retained-allele dose in hematologic cohorts: hazard ratios for multi-hit versus single-hit TP53 mutation in the two AML cohorts and in the MDS (IPSS-M) cohort; only the large MDS cohort reaches significance. (**b**) Allelic state in solid tumors: hazard ratios for each state versus retained missense-only, estimated within tumor type by Cox regression (adjusted for age where available) and pooled across types by inverse-variance weighting; no state separates from retained missense-only. The two panels ask the same question in the two compartments: a dose effect in blood, none in solid tumors.

## Data Availability

This study re-analyzed publicly available datasets and generated derived data: the raw inputs are public, and the data produced by our analysis (derived result tables and figures) are provided in the Supplementary Materials and in the analysis repository. Mutation, clinical and discrete copy-number data were retrieved through the cBioPortal REST API (https://www.cbioportal.org/api accessed on 24 September 2026) for the studies listed in the retrieval manifest, and the independent validations used the NCI TP53 Database release r21 (https://tp53.cancer.gov/ accessed on 24 September 2026) and COSMIC release v104, GRCh38 (Mutant Census, Sample and Classification files; https://cancer.sanger.ac.uk/cosmic, accessed 24 September 2026), the latter used under the terms of the COSMIC licence with the citation it requires. Because these repositories are updated continuously, the exact input set analyzed here is preserved as retrieval snapshots (retrieved 4–22 September 2026, plus the COSMIC release v104 files registered on 24 September 2026) shipped with a machine-readable manifest giving the SHA-256 checksum, byte size, record count, source and endpoint of every retrieved file (results/retrieval_manifest.csv; see also REPRODUCIBILITY.md), together with the complete analysis pipeline and all derived tables and figures. The snapshots verify offline and can be re-derived live; the bundle is available from the corresponding author and will be deposited in a public repository (Zenodo, with DOI) upon acceptance.

## Supplementary Materials

The following are available online at https://www.mdpi.com/article/10.3390/ijms272110024/s1.

## Abbreviations

The following abbreviations are used in this manuscript: CHIP: clonal hematopoiesis of indeterminate potential; GOF: gain of function; WES: whole-exome sequencing; WGS: whole-genome sequencing; TCGA: The Cancer Genome Atlas; NCI: National Cancer Institute; IARC: International Agency for Research on Cancer; HR: hazard ratio; CI: confidence interval; IPSS-M: Molecular International Prognostic Scoring System; MDS: myelodysplastic syndromes; AML: acute myeloid leukemia; CLL: chronic lymphocytic leukemia; DLBCL: diffuse large B-cell lymphoma; ALL: acute lymphoblastic leukemia; COSMIC: Catalogue Of Somatic Mutations In Cancer; GISTIC: Genomic Identification of Significant Targets In Cancer; LOH: loss of heterozygosity; CNA: copy-number alteration; TCR: T-cell receptor; HLA: human leukocyte antigen.
